# Genetic variants in microRNA genes: impact on microRNA expression, function, and disease

**DOI:** 10.3389/fgene.2015.00186

**Published:** 2015-05-21

**Authors:** Sophia Cammaerts, Mojca Strazisar, Peter De Rijk, Jurgen Del Favero

**Affiliations:** ^1^Applied Molecular Genomics Unit, Department of Molecular Genetics, VIB, University of AntwerpAntwerp, Belgium; ^2^Multiplicom N.V., NielBelgium

**Keywords:** microRNA, genetic variants, expression, function, disease

## Abstract

MicroRNAs (miRNAs) are important regulators of gene expression and like any other gene, their coding sequences are subject to genetic variation. Variants in miRNA genes can have profound effects on miRNA functionality at all levels, including miRNA transcription, maturation, and target specificity, and as such they can also contribute to disease. The impact of variants in miRNA genes is the focus of the present review. To put these effects into context, we first discuss the requirements of miRNA transcripts for maturation. In the last part an overview of available databases and tools and experimental approaches to investigate miRNA variants related to human disease is presented.

## Introduction

miRNAs are short, non-protein-coding RNA molecules that mediate post-transcriptional regulation by affecting mRNA stability and translational repression or activation (Vasudevan et al., 2007; Filipowicz et al., 2008).

miRNAs were first discovered in 1993 in *Caenorhabditis elegans* (Lee et al., 1993). In 2000–2001 many other miRNA genes were identified in *C. elegans* and miRNAs were shown to be widely present in other species (Pasquinelli et al., 2000; Lagos-Quintana et al., 2001; Lau et al., 2001; Lee and Ambros, 2001). Since then, given their impact on expression, miRNAs have been extensively studied and the miRBase database, the central miRNA sequence repository, continues to expand, collecting novel miRNA genes in a wide variety of species (Kozomara and Griffiths-Jones, 2014). Even in the human genome, with 1881 precursors and 2588 mature miRNA sequences deposited in miRBase version 21, many novel miRNA genes are being identified and published continuously (Friedländer et al., 2014; Cheng et al., 2015; Londin et al., 2015). Despite the extensive research, we are still uncovering aspects of the miRNA maturation process and we have to date only a limited understanding of the functions of specific miRNAs.

miRNAs add a layer of complexity to gene regulation. They target transcripts mainly by complementarity to the seed region, nucleotides (nt) 2–7 of the mature miRNA molecule (Bartel, 2009). This minimal requirement for complementarity results in targeting of many mRNAs by a single miRNA and targeting of one mRNA by several miRNAs (Lim et al., 2005; Peter, 2010; Wu et al., 2010). In addition, miRNAs can have sequence and length variability (isomiRs), potentially resulting in altered targeting capacity and/or specificity. One of the aspects that further influences the complexity of the miRNA repertoire are genetic variants.

This review provides an overview of the current knowledge of the influence of genetic variants on miRNA biogenesis and function. First, miRNA biogenesis and requirements of the miRNA processing enzymes are described, followed by a discussion of isomiRs and their functional implications. In the second part, we highlight the effect of genetic variants on the expression and functioning of miRNAs. In the last part, approaches that are used to identify miRNAs involved in diseases are discussed, with a focus on genetic strategies. Furthermore, we present available tools, databases, and experimental approaches to aid functional characterization studies of disease-associated miRNA variants.

## MicroRNA Biogenesis and Generation of IsomiRs

miRNA genes can be classified in several categories according to their location in the genome. A majority of the currently known human miRNA genes deposited in miRBase are intergenic (68%). Of the intragenic miRNAs, most are intronic (12% of all genes). The remaining genes are located in repeats, lncRNAs, UTRs, or coding regions of host genes (Londin et al., 2015). miRNA genes are often located close to other miRNA genes in so-called clusters. miRNAs located in host genes or clustered with other miRNAs can be cotranscribed, which is supported by good correlation of their expression patterns for several genes (Baskerville and Bartel, 2005). Alternatively, intronic miRNAs can be transcribed independently from their host gene and polycistronic miRNA transcripts can undergo alternative splicing to yield specific miRNA expression (Ramalingam et al., 2014).

In the canonical biogenesis pathway, primary miRNA transcripts (pri-miRNAs) are cleaved in the nucleus by the Microprocessor complex, a complex consisting of ribonuclease Drosha and its cofactor DGCR8, to release the shorter precursor miRNA (pre-miRNA). This precursor molecule is transported to the cytosol by the nucleocytoplasmic transport protein Exportin-5, where it is cleaved by the ribonuclease Dicer to result in a mature miRNA duplex consisting of the mature 5p and 3p strands. The duplex is loaded onto an Argonaute protein, the core component of the RNA induced silencing complex (RISC). One of the strands is discarded, while the other mediates posttranscriptional regulation by base pairing to target mRNAs.

There are different types of target sites. Canonical sites for miRNA targeting are seed matches (nt 2–7 of the mature miRNA) with an adenine opposite from the first nt of the mature miRNA and/or complementarity to the eighth nt of the miRNA. This seed match can be supplemented with complementarity to nt 13–16 (3′ supplementary sites). Extended sites of complementarity at the 3′ end of the miRNA can also compensate for seed mismatches (3′ compensatory sites) (Bartel, 2009). Though a majority of the miRNA-target interactions include seed matches, many non-seed interactions are also observed (Helwak et al., 2013). Similarly, though mRNAs are the predominant miRNA targets, 30% of all miRNA targets are other classes of RNA, such as rRNA, tRNA, snRNA, miRNA, lincRNA, and pseudogenes (Helwak et al., 2013).

Mirtrons, miRNAs encoded within short introns of host genes, bypass processing by the Microprocessor complex. By splicing the mirtron is released and can be, if needed after 5′ or 3′ tail trimming, transported to the cytosol to be further processed by Dicer. In the study of Ladewig et al. (2012), 240 human mirtrons were identified, indicating that bypassing Drosha is not a rare phenomenon. Other non-canonical miRNA biogenesis pathways have also been discovered and were recently reviewed by Abdelfattah et al. (2014). In the next sections, we will focus on the general miRNA characteristics and substrate requirements of the key enzymes of the canonical miRNA maturation pathway.

### Maturation Enzyme Requirements

#### Microprocessor Complex

Drosha, a nuclear ribonuclease, is the enzyme that cleaves the miRNA hairpin from the primary transcript, whereby it generates a precursor with 3′ end overhangs of 2 nt (Lee et al., 2003). An essential cofactor for its pri-miRNA processing activity is the protein DGRC8. The complex containing Drosha and DGCR8, also called the Microprocessor complex, is sufficient for pri-miRNA processing (Denli et al., 2004; Gregory et al., 2004; Han et al., 2004; Landthaler et al., 2004). The capacity of DGCR8 to bind pri-miRNAs is necessary for Drosha cleavage (Yeom et al., 2006). DCGR8 trimerizes upon binding of pri-miRNAs and formation of this protein-pri-miRNA complex may allow specific recognition of pri-miRNAs as opposed to other types of RNA and trigger subsequent cleavage by Drosha (Faller et al., 2010).

Several common features of pri-miRNAs were determined by thermodynamic profiling of secondary structure predictions on a set of human and fly pri-miRNAs: the transcript contains a local hairpin structure with an imperfectly paired stem of ~33 base pairs (bp), consisting of an upper stem (~22 bp, region of the mature miRNA duplex) and a lower stem (~11 bp), a terminal loop region and flanking segments that are usually single-stranded or have large bulges or internal loops (Han et al., 2006). Essential requirements of pri-miRNAs for Drosha processing were experimentally determined. The stem-loop structure within the pri-miRNA needs to be larger than the pre-miRNA: it needs an extended stem (i.e., the lower stem, ~11 bp) and an unstructured region flanking the hairpin (Lee et al., 2003; Chen et al., 2004; Zeng and Cullen, 2005; Zeng et al., 2005; Han et al., 2006; Auyeung et al., 2013). *In vitro* cleavage assays showed that Drosha was able to process pri-miR-30a and pri-miR-16-1 with as little as ~20–25 nt of flanking regions (Lee et al., 2003; Han et al., 2004). However, for correct *in vivo* processing longer flanking regions are required (Chen et al., 2004). The presence of a large unstructured terminal loop region is beneficial for Drosha processing, as reduction of the size of the predicted terminal loop results in reduced processing (Zeng et al., 2005; Han et al., 2006; Zhang and Zeng, 2010). These properties correspond with the *in silico* determined characteristics of known miRNA hairpins.

Two different models have been proposed to explain how Drosha determines where to cleave the pri-miRNA. One study proposed that the Microprocessor mainly determines the distance from the terminal loop region of the hairpin and cleaves ~22 bp upstream (Zeng et al., 2005). Another hypothesis proposed that Drosha predominantly cleaves ~11 bp away from the junction between the lower hairpin stem and the flanking regions (Han et al., 2006). A recent study showed that the complex determines the distance to both ssRNA–dsRNA junctions and that both distances need to be optimal in order to result in precise cleavage (Ma et al., 2013).

Next to the structural prerequisites of the hairpin, position-specific sequence motifs present in the hairpin flanking segments of the primary transcript (UG motif, CNNC motif) and in the loop region (UGU or GUG motif) can enhance processing efficiency in human cells. Nearly 80% of the human pri-miRNAs that are conserved between human and mice contain at least one of these motifs (Auyeung et al., 2013).

#### Exportin-5

The transport of pre-miRNAs from the nucleus to the cytosol is mediated by Exportin-5 in the presence of cofactor RanGTP (Yi et al., 2003; Bohnsack et al., 2004; Lund et al., 2004). The RNA structure is the main determinant for precursor binding: a stem of at least 18 bp and a blunt end or a 3′ overhang, such as the overhang created by Drosha (Lee et al., 2003), is preferential (Zeng and Cullen, 2004). Upon binding, the 3′ 2 nt overhang of the precursor and a large part of the stem is bound by Exportin-5-RanGTP in a sequence-independent manner, which protects the precursor from degradation by nucleases (Bohnsack et al., 2004; Zeng and Cullen, 2004; Okada et al., 2009).

#### Dicer

Dicer, a ribonuclease that processes dsRNA into duplexes with a length of ~22 nt, processes pre-miRNAs into mature miRNA duplexes (Bernstein et al., 2001; Grishok et al., 2001; Hutvágner et al., 2001; Ketting et al., 2001; Knight and Bass, 2001). Dicer cleaves its substrates gradually with a preference to start at the termini of the RNA duplex and generates 2 nt 3′ overhangs (Zhang et al., 2002, 2004). The structure of the termini contributes to the size of the end product, with 2 nt 3′ overhangs resulting in products less than 24 nt, while blunt ends result in longer fragments (Vermeulen et al., 2005). Dicer efficiency is affected by several substrate parameters, such as the size of the loop (in case of pre-miRNAs) and the sequence and the size of the overhangs, with 3′ overhangs of 2 nt being the most efficient substrate for human Dicer (Vermeulen et al., 2005; Lund and Dahlberg, 2006; Zhang and Zeng, 2010; Park et al., 2011). Dicer binds both the 5′ and 3′ end of the precursor molecule and mainly cleaves at a distance of 22 nt from the 5′ end (Park et al., 2011). The accuracy of the cleavage seems to depend on the distance between this canonical cleavage site and the nearest bulge or the terminal loop (Gu et al., 2012). The Drosha-mediated generation of pre-miRNAs with a 3′ overhang of 2 nt and a stem of ~22 bp thus provides suitable substrates for Dicer. This results in the removal of the loop by Dicer and acquirement of mature miRNA duplexes of ~22 nt bearing 2 nt overhangs at the 3′ end of both strands, on one side generated by Drosha and at the other side generated by Dicer.

#### Argonaute

The mature miRNA duplex is loaded onto an Argonaute protein by the RISC loading complex. This complex matures to RISC when one of the strands, termed the passenger strand, is removed (Czech and Hannon, 2011). The other strand, termed the guide strand, can then, in complex with Argonaute, target RNAs via sequence complementarity for posttranscriptional regulation. The process of strand selection is asymmetric: for many miRNAs one of the strands is preferentially retained. This bias is in part explained by the relative stability of the 5′ end of both strands, where the strand with the lowest stability is preferentially retained within RISC (Khvorova et al., 2003; Schwarz et al., 2003). Another contributing factor is the sequence composition of the strands: for human miRNA duplexes with a large difference in expression between both strands, the highest expressed strand has a bias toward a uracil at position 1 and a high purine content, while the lower expressed strand has a bias toward a cytosine at position 1 and a high pyrimidine content (Hu et al., 2009). However, as the dominant strand can vary between tissues (Cloonan et al., 2011), other, yet unknown, factors than sequence and structure of the miRNA duplex itself must be contributing to this variable selection bias.

### Proteins Involved in Regulation of Biogenesis

In addition to the biogenesis key proteins described above, a number of other proteins are known to be involved in the regulation of miRNA biogenesis. The following examples highlight the relevance of the terminal loop region in this regulation. The first example is the protein KSRP. This protein binds directly to the terminal loop of a set of miRNAs and enhances their processing by Drosha and Dicer (Trabucchi et al., 2009). Another example is the regulation by Lin28 and terminal uridyl transferase TUTase4. Lin28 binds a GGAG sequence motif in the terminal loop of specific pre-miRNAs, including let-7 family members, upon which TUTase4 binds to the Lin28-pre-miRNA complex and uridylates the 3′ end of the pre-miRNA. This oligo-uridylation prevents processing by Dicer (Heo et al., 2008, 2009; Hagan et al., 2009). In addition to the Lin28-mediated inhibitory role of TUTase4, this enzyme (and other terminal uridyl transferases) also mono-uridylates a subset of pre-let-7 family members and other miRNAs with a 3′ 1 nt overhang, promoting Dicer processing (Heo et al., 2012). An overview of all proteins involved in the regulation of miRNA biogenesis can be found in Siomi and Siomi (2010).

### IsomiR Repertoire

Mature miRNAs originating from one arm of a pre-miRNA can have sequence and length heterogeneity *in vivo*, with sequences and lengths closely related to, but different from the canonical miRNA sequence reported in miRBase. Such alternative sequences are termed isomiRs (Morin et al., 2008). Different types of isomiRs have been observed: templated additions or deletions at the 5′ and/or 3′ end of the miRNA, non-templated additions at the 3′ end and substitutions within the sequence (Landgraf et al., 2007; Morin et al., 2008; Martí et al., 2010). Alternative processing by Drosha or Dicer, Dicer-independent Argonaute2-mediated cleavage and exonuclease degradation of the 3′ terminus of miRNAs can be a source of templated isomiR genesis. Non-templated changes can be established by nucleotidyl transferases, posttranscriptional editing or due to the presence of genetic variants within the transcript (Morin et al., 2008; Cloonan et al., 2011; Neilsen et al., 2012; Lee et al., 2013; Ma et al., 2013).

Since miRNAs target transcripts via imperfect sequence complementarity, isomiRs with a changed sequence for the nucleotides contributing to the target specificity are expected to affect the target spectrum of the miRNA. Given that the majority of miRNA-target interactions include matches to the miRNA seed (Helwak et al., 2013), isomiRs with an altered seed sequence, such as 5′ isomiRs, may have a large influence on target specificity. Sets of isomiRs with an altered seed sequence were predicted to gain the capacity to regulate many additional genes and/or lose the capacity to regulate a subset of genes compared to the canonical miRNA (Gong et al., 2012; Tan et al., 2014). Gain or (partial) loss of function of isomiRs with altered seed sequence and even of isomiRs with a preserved seed has been confirmed experimentally (Kawahara et al., 2007; Gong et al., 2012; Chan et al., 2013; Tan et al., 2014). The size of the effect will, however, be largely determined by the absolute and relative expression and stability of the isomiR compared to the canonical miRNA *in vivo* and its binding efficiency to RISC, which can also be affected by the sequence variability (Chan et al., 2013; Llorens et al., 2013). Alternatively, it has been suggested that isomiRs, which often show a high expression correlation with their canonical miRNAs, may function to regulate the same pathways as the canonical miRNA and that the sequence heterogeneity within isomiR populations may act to reduce off-target effects (Cloonan et al., 2011). The broader functional implications of isomiRs, their contribution to gene expression regulation and how their expression is controlled remains to be further elucidated.

## Impact of Genetic Variants on miRNA Expression and Function

As presented above, miRNA transcripts need to fulfill structural and sequence prerequisites in order to result in expression of the correct mature miRNA sequence(s). Sequence variability in miRNA genes can therefore influence both the expression level as the functionality of the miRNA and consequently will result in differential regulation of their target genes.

Large-scale *in silico* analyses of single nucleotide polymorphisms (SNPs) in human miRNA genes have demonstrated that miRNA genes have lower SNP densities than their flanking regions or the human genome (Saunders et al., 2007; Gong et al., 2012; Han and Zheng, 2013). Within the miRNA gene the mature sequence has a lower SNP density than the precursor, with the seed having the lowest SNP density, reflecting their functional importance. In addition, it was shown that there is a negative correlation between the number of SNPs a miRNA gene harbors and the number of diseases the miRNA is associated with (Han and Zheng, 2013). This again highlights the importance of genetic variants in miRNA genes related to their function and their involvement in human diseases.

Genetic variants can affect miRNAs on several levels. Variants in miRNA promoter regions and other regulatory regions may result in an altered transcription rate. Variants in splice sites of the host gene (for intronic miRNAs) or of the polycistron (clustered miRNAs) could result in aberrant expression patterns. Next, variants within the miRNA transcript can have an effect on miRNA maturation in multiple aspects. They can change the binding affinity of the miRNA hairpin to biogenesis enzymes or accessory proteins (sequence motifs or structural motifs that are changed due to the modified underlying sequence). Variants can lead to altered processing accuracy or to changed frequency of alternative cleavage sites of biogenesis enzymes. They can also lead to altered strand loading bias into RISC. These changes in maturation can all result in altered expression of the canonical miRNA and its existing isomiRs, resulting in deregulation of target genes. It may also result in production of novel isomiRs, which can lead to altered functionality of the miRNA. Lastly, genetic variants within the mature sequence can affect target specificity by generating isomiRs.

Given the potentially huge impact of genetic variants on the tightly regulated miRNA repertoire, and the importance of miRNA-mediated gene regulation, it is not surprising that genetic variants have been found to be causal for or associated with human diseases (Mencía et al., 2009; Hughes et al., 2011). Variants in miRNA biogenesis enzymes and in miRNA binding sites can also lead to impaired miRNA regulation. However, the latter category of variants would likely have a smaller impact than the former two categories, because only the targeting of one gene by one miRNA would be disrupted in case the target does not contain multiple binding sites for that miRNA. Genetic variants in miRNA genes, biogenesis genes and target binding sites associated with human diseases were recently reviewed by Kawahara (2014). Here, we will focus on illustrating the different effects genetic variants can have on miRNA transcription, maturation, and targeting with examples from human miRNA variants studied in disease where possible.

### Variants Influencing miRNA Transcription or Splicing

Though bio-informatics approaches have been used to predict miRNA gene promoters (Saini et al., 2007; Marsico et al., 2013), many miRNA gene promoters have not yet been experimentally validated. Below we describe an example of a variant in a miRNA promoter, which was experimentally delineated. The second example is a case of a variant in a host gene promoter for an intronic miRNA cluster. However, experimental investigation of this type of variants can be complicated by the presence of an independent miRNA promoter in addition to the host gene promoter.

SNP rs57095329 is located in the promoter region of MIR146A, 17 kb upstream of the pre-miR-146a sequence. This variant was found to be associated with systemic lupus erythematosus (SLE). The risk allele reduces the binding to transcription factor Ets-1, another SLE susceptibility gene, and results in decreased promoter activity. Consistent with this mode of action, risk allele carriers have lower expression of miR-146a-5p (Luo et al., 2011).

SNP rs999885 is located in the promoter region of the protein-coding gene MCM7. This variant was associated with a decreased risk of chronic hepatitis B infection, but also with an increased risk of hepatocellular carcinoma (HCC) in individuals with chronic hepatitis B virus infection. The miR-106b-25 cluster, coding for miR-106b, miR-93, and miR-25, is located in the 13th intron of MCM7. HCC patients that carry the risk allele have higher expression of the miRNA transcript in non-tumor liver tissue than non-carriers (Liu et al., 2012). Recently it was shown that this cluster can be transcribed independently of MCM7 via different promoters and that the miRNA polycistronic transcript can undergo alternative splicing (Ramalingam et al., 2014). Therefore it would be very interesting to uncover whether and how this variant influences the relative and absolute expression of the different mature miRNAs in this cluster.

To the best of our knowledge, variants in splice sites that affect miRNA expression in human disease have not been published yet. Nevertheless, the effect of host gene splice variants on mature miRNA expression has been demonstrated by mutagenesis experiments (Janas et al., 2011).

### Variants Influencing miRNA Maturation

Variants within the pri-miRNA can affect miRNA expression levels by inducing changes in the maturation process, as described above. This effect can take place at several steps in the maturation pathway: Drosha processing, Dicer processing, and/or altering strand preference.

An example of a variant with and effect on the Drosha processing step is rs2910164. This variant is located in the seed sequence of miR-146a-3p and was predicted to lead to a mismatch in the hairpin stem. The heterozygous variant genotype was associated with increased risk of papillary thyroid carcinoma (PTC). After overexpression of pri-miR-146a with the G allele or the C allele in cells, the C allele resulted in nearly twofold reduction of pre-miR-146a compared to the G allele. The reduced production of pre-miR-146a from pri-miR-146a with a C allele was also confirmed by an *in vitro* processing assay (Jazdzewski et al., 2008). An example of a pri-miRNA variant affecting the first processing step is a variant located four bases upstream of the miR-510-5p sequence. This variant was identified in a study screening X-linked miRNA genes in male schizophrenia patients and control individuals. The variant was predicted to alter the secondary structure around the Drosha processing site. Functional validation showed that the variant results in increased expression of pre-miR-510, miR-510-5p, and miR-510-3p (Feng et al., 2009; Sun et al., 2009).

A variant affecting Dicer processing is rs546098287, located within the seed sequence of miR-96-3p. This variant was identified as the causative mutation in an Italian family with non-syndromic hearing loss (Soldà et al., 2012). Two segregating point mutations in miR-96-5p were previously identified in two Spanish families with non-syndromic hearing loss, demonstrating the importance of this gene in the disease pathogenesis (Mencía et al., 2009). The mutation in miR-96-3p reduces the processing from pre-miR-96 to both miR-96-5p and miR-96-3p. This is likely induced by a structural change, because the variant was predicted to enlarge an internal loop in the hairpin stem and when a second variant was introduced to restore the original stem structure, the expression was largely brought back to normal levels (Soldà et al., 2012).

An example of a variant influencing the relative strand abundance is a variant in MIR133A2, identified in a patient with atrial fibrillation. The variant is located at the 3′ end of the miR-133a-3p sequence and was predicted to alter the secondary structure of the hairpin at the base of the miR-133a duplex. miR-133a was found to be highly expressed in atrial tissue, with the 3p strand being the dominant one, while miR-133a-5p represented less than 1% of all the miR-133a reads. Functional validation experiments demonstrated that the variant resulted in increased miR-133a-5p expression, but had no significant effect on miR-133a-3p expression. The variant thus increases the relative abundance of the 5p strand compared to the 3p strand (Ohanian et al., 2013).

The cases described above are all variants located either within the precursor sequence or close to it. A study by Diederichs and Haber (2006) reported the functional validation of 15 pri-miRNA variants ranging from 5 to 133 nt outside the pre-miRNA sequence, identified in cancer cell lines. Despite some of these variants had predicted structural changes at or near the base of the miRNA hairpin, none of the variants displayed detectable differences in mature miRNA expression. On the other hand, for the variant rs11134527, which is located 96 nt outside the MIR218-2 hairpin sequence, it was shown that it does change the expression of miR-218-5p significantly (Gao et al., 2013). Therefore variants located further outside the hairpin can still affect miRNA biogenesis, although variants closer to the hairpin are more likely to have a higher impact.

### miRNA Variants Influencing Targeting

Beside resulting in altered expression, miRNA variants can also result in altered target specificity by creating isomiRs.

An interesting example of a variant creating a polymorphic isomiR is rs2910164 in miR-146a-3p. This variant also affects Drosha processing as described above (Jazdzewski et al., 2008). The isomiR and canonical miR-146a-3p are predicted to share only a small set of target genes. Transcriptome analysis in healthy and in tumor tissue of PTC patients with GG or GC genotype showed that hundreds of genes (358 in unaffected tissue, 575 genes in tumor tissue) were differentially expressed (Jazdzewski et al., 2009). Even though this does not provide direct evidence that all those genes are direct targets of the isomiR, it does give insight in the large-scale downstream changes isomiRs may induce.

Direct evidence of altered targeting was shown in a study investigating the effect of eight seed SNPs and three mature sequence SNPs (among which one deletion) identified in dbSNP in nine miRNA genes. Four of the tested miRNAs (miR-627-5p, miR-379-5p, miR-499-3p, and miR-124-3p) partially or completely lost their potential to suppress their original target due to a SNP in their seed sequence. The seed sequence variant rs2620381 in miR-627-5p not only resulted in loss of targeting of SEMA3F, but also in gained capacity to suppress ATP6V0E1 (Gong et al., 2012). In addition, miR-379-5p, miR-940, and miR-34a-5p containing variants in the mature sequence also displayed partial or total loss of function of their original mRNA targets tested.

## Studying miRNAs Involved in Disease

### miRNA Profiling and Genetic Studies

To study if and which miRNAs play a role in the pathogenesis of a disease, two main strategies can be used: a miRNA profiling approach and a genetic approach.

In the miRNA profiling approach, the affected tissue of a group of patients is subjected to miRNA expression profiling and compared to the expression profile of either the same tissue in healthy individuals, tissue of patients with a different disease or subphenotype, or to adjacent non-affected tissue of the same patients (Calin et al., 2005; Kan et al., 2012; Feliciano et al., 2013; Huang et al., 2014). The tested miRNAs can range from selected candidate miRNAs to a genome-wide approach and different technologies are available, such as RT-qPCR, microarray based methods or RNA sequencing (Pritchard et al., 2012). The miRNA expression profiles are compared between the disease phenotype and the controls in order to determine whether and which miRNAs are differentially expressed. Resulting signatures may be used as diagnostic or prognostic biomarkers (Calin and Croce, 2006) and can provide clues about the role of miRNAs in the disease mechanism. Additionally, interesting miRNAs can be further investigated to dissect their mode of action.

In the genetic approach, the starting point is a linkage or an association analysis (genome-wide or candidate gene approach) including but not necessarily limited to miRNA genes between patients and control individuals (Mencía et al., 2009; Schizophrenia Psychiatric Genome-Wide Association Study (GWAS) Consortium, 2011; Zhang et al., 2015). When a genetic variant in or near a miRNA gene is associated with or is causal for the disease, functional validation can be initiated to assess its effect and its role in the pathogenesis of the disease. Variants in miRNA genes linked to the disease could also be used for disease diagnosis, as exemplified by the patent on rare miRNA gene variants identified in schizophrenia patients (Sommer and Rossi, 2012). The genetic screening approach focused on miRNA genes is not yet as frequently applied on a large scale as genome-wide miRNA profiling.

Both strategies have limitations and benefits. General considerations regarding miRNA profiling were reviewed by Pritchard et al. (2012). In the study of disease, genome-wide miRNA profiling can provide a global insight in which miRNAs may play a role in the disease by generating disease-specific miRNA expression signatures. An advantage of this approach is that the deregulated miRNA provides a first indication of its possible functional role in the disease. The main limitation, however, is that the differential expression of a miRNA in itself does not provide an indication of causality: the altered expression can be a causal factor, a consequence, or could even be unrelated to the disease pathogenesis (Kan et al., 2012). Another constraint is that the affected tissue has to be accessible. This is especially problematic in the study of neurological diseases. Alternatively, brain tissue of deceased patients and healthy individuals, if available, can be used. However, though many miRNAs seem to be relatively stable (Bail et al., 2010; Winter and Diederichs, 2011), the half-lives of some miRNAs are less than 1 hour (Sethi and Lukiw, 2009). This implicates that when brain samples with different postmortem delay from death to tissue processing are compared, variability may arise due to differential instability of the miRNAs in the samples. The restricted access to postmortem brain samples can also result in suboptimal sample sizes. A much more accessible proxy for unavailable affected tissues is blood. While this may provide a good avenue for determining diagnostic or prognostic biomarkers, it is not clear whether the miRNAs deregulated in blood would also be deregulated in the affected tissue. In addition, several factors can influence miRNA expression in blood, such as age, drug use, and other diseases (Margis et al., 2011; de Boer et al., 2013; Meder et al., 2014).

The main advantage of a genetic approach is that when a variant is associated with disease risk, it gives an indication that the miRNA involved has a primary effect in the disease pathomechanism (large or small contributing effect), which cannot be determined by expression profiling. This advantage also has a downside: while the association or linkage analysis may indicate a miRNA variant is likely to be involved in the disease, it is not at all clear whether the identified variant has a functional effect and whether the expression of that miRNA is altered in the disease state. Another asset of the genetic approach is that diseases for which the main affected tissue is not accessible can still be studied because genomic DNA can be isolated from more accessible patient material, such as blood or saliva. There is, however, still a chance of missing a mutation, if the patient is mosaic for the mutation and the load of the mutation is lower in the examined tissue than in the affected tissue.

Both approaches are complementary and should both be used to determine the involvement of a miRNA in relation to a disease: genetic approaches can pinpoint the causal or risk factors involved in the disease, while expression studies reveal whether and how the expression of the disease-associated miRNAs is altered in the affected tissue. Neither approach is sufficient to determine both. An example of a study combining both approaches is the study of Calin et al. (2005) where miRNA profiling in affected tissue of patients was performed, followed by variant screening in a set of relevant miRNA genes. An expression signature of thirteen miRNA genes was found to be able to distinguish between patient subgroups. In two of these genes, patient-specific variants were found. One of the variants was a functional germline mutation in pri-miR-15a/16-1. On the other hand, when a genetic variant is associated with a disease, usually only the expression of that particular miRNA is assessed in patient material. The profiling and genetic approach has also been combined on a large scale by miRNA profiling in blood samples of patients with different diseases and investigating whether the deregulated miRNA genes were located within 250 kb of published GWAS significant SNPs (Keller et al., 2011). While on average 103 deregulated miRNAs were identified in each disease, of all the deregulated miRNAs they found only six miRNA genes in proximity to a GWAS hit associated with the same disease. Combining the genetic and the profiling approach on the same set of patients could thus provide an invaluable amount of information on both DNA and RNA level from which the disease-contributing miRNAs can be separated from the miRNAs that are probably deregulated due to secondary disease processes.

### Databases and Tools for Genetic Approaches

To provide direct evidence that a genetic variant in or near a miRNA gene and the deregulated miRNA expression in disease tissue are linked, detailed functional validation is required. Functional validation is labor-intensive, costly, and time-consuming, therefore researchers usually prioritize the potential candidate variants using *in silico* approaches. In this section, we will focus on available databases and tools for the impact prediction of genetic variants on miRNA function (**Tables 1** and **2**). Different types of resources for miRNA research have been reviewed by Vlachos and Hatzigeorgiou (2013).

**Table 1:** Overview of described databases.

Name	Use	URL
miRBase	Database for miRNA sequences and gene annotations	http://www.mirbase.org/
miRNASNP	Database for miRNA gene variants, location annotation, RNA secondary structure impact prediction, target impact prediction for seed variants	http://bioinfo.life.hust.edu.cn/miRNASNP2/index.php
miRNA SNiPer	Database for miRNA gene variants, location annotation	http://www.integratomics-time.com/miRNA-SNiPer/
miRvar	Database for miRNA gene variants, location annotation, predicted impact on Dicer and RISC loading	http://genome.igib.res.in/mirlovd/home.php
PolymiRTS Database	Database with miRNA seed variants and predicted effect on targeting	http://compbio.uthsc.edu/miRSNP/
dPORE-miRNA	Database with miRNA promoter variants and their predicted effect on transcription binding	http://cbrc.kaust.edu.sa/dpore/

*The Use column refers to the usage described in the text. Most of the databases also incorporate other information not mentioned here.*

**Table 2:** Overview of described software.

Name	Use	URL
miRVaS	Location annotation, RNA secondary structure impact prediction for miRNA gene variants	http://mirvas.bioinf.be/
Mfold	RNA secondary structure prediction	http://mfold.rna.albany.edu/
RNAfold	RNA secondary structure prediction	http://rna.tbi.univie.ac.at/cgi-bin/RNAfold.cgi
PHDcleav	Prediction of Dicer processing sites	http://www.imtech.res.in/raghava/phdcleav/
RISC binder	Prediction of preferential strand loaded into RISC	http://crdd.osdd.net:8081/RISCbinder/

A first step in the assessment of the relevance of a genetic variant or a set of genetic variants is determining whether the variant is located near or in a specific region of a miRNA gene. For a single variant of interest, the variant location may be assessed in a genome browser to determine the position of the variant compared to neighboring miRNA genes. To evaluate the exact location of the variant relative to the different miRNA gene regions (e.g., seed, mature, terminal loop), one also has to compare this location with the information provided about the gene in miRBase (Kozomara and Griffiths-Jones, 2014). This procedure is tedious and error-prone, especially when investigating multiple miRNA variants. For variant lists such as those derived from massively parallel sequencing projects, the first annotation step can be done with any of the annotation tools available to determine which (miRNA) genes, if any, are located near the variants.

Online repositories compiling information about known SNPs in miRNA genes, such as miRNASNP (Gong et al., 2012), miRNA SNiPer (Zorc et al., 2015), and miRvar (Bhartiya et al., 2011), can be used to extract exact location information for these variants relative to the miRNA hairpin. A limitation for the last two databases is that the search needs to be initiated by using the miRNA gene name instead of the SNP ID. In addition, databases cannot be used when investigating novel variants and are less suitable when investigating multiple variants in several miRNA genes. To eliminate the time-consuming processes related to assessing the position of variants in the context of miRNA genes and what their predicted structural impact is, we recently developed the freely available software miRVaS^1^. Required input is the genomic location of a (known or novel) variant or of a list of variants.

The next possible step in determining the potential relevance of a genetic variant is predicting its effect on the secondary structure of the miRNA. For known SNPs within miRNA genes, structure prediction results of the miRNA hairpin with and without the variant can be found in the miRNASNP database (Gong et al., 2012). The difference in free energy is calculated and a predicted effect on the miRNA expression is provided, based on the assumption that variants destabilizing the hairpin will reduce the expression and stabilizing variants will increase the expression of the mature miRNA (Gong et al., 2012). For novel variants the RNA secondary structures can be predicted for wild type and variant sequences using web-based RNA structure prediction tools, such as the Mfold or RNAfold web servers (Zuker, 2003; Gruber et al., 2008), and compared. Again, this process is time-consuming and not suitable for the analysis of large variant sets. miRVaS^1^ automates this type of analysis, predicting structural impact for lists of variants, needing only the genomic location and alternative allele of the variant.

If a variant is located in a miRNA seed sequence, the effect on target binding can also be assessed *in silico*. Both PolymiRTS database (Bhattacharya et al., 2014) and miRNASNP (Gong et al., 2012) contain lists of predicted gain and loss of target binding sites for seed sequence variants that can be browsed. To assess the impact of variants in miRNA promoter regions on transcription factor binding, the dPORE-miRNA database can be queried (Schmeier et al., 2011). Algorithms to predict Dicer cleavage patterns and preferential strand loading into RISC, such as PHDcleav and RISC binder (Ahmed et al., 2009, 2013) could also be useful to assess the impact of variants. The predicted effect on Dicer cleavage and RISC binding induced by variants is incorporated in the miRvar database (Bhartiya et al., 2011).

### Functional Characterization of a Disease-Associated miRNA Variant

Using tools and databases, a set of genetic variants can be prioritized based on their potential functional relevance. The most promising candidates can then be chosen for direct functional validation in cell-based assays. The assays used depend on the location of the variant and the hypothesis.

For variants in miRNA promoter regions, the wild type and variant promoter can be cloned into a promoter-less reporter gene vector and transfected (transiently or stably) into cell lines to assess whether there is a difference in promoter activity due to the variant and whether the variant promoter has different binding affinity to transcription factors (Luo et al., 2011).

To assess the impact of a genetic variant on miRNA biogenesis and/or targeting, cell lines overexpressing wild type and mutant miRNAs are usually established. The constructs should include the full precursor gene and upstream and downstream flanking sequences. The inclusion of flanking regions is crucial: the biogenesis machinery in the cell may not efficiently recognize or process the transcript when the flanking regions are absent or too small, as evidenced by the study of Chen et al. (2004) where only constructs for miR-223 with at least 40 nt of flanking region resulted in detectable miR-223 expression. Using up- and downstream flanks of 125 nt resulted in mature miRNA expression for all 13 tested miRNAs in this study, showing that the incorporation of longer flanks in miRNA constructs is generally a good strategy. To assess the effect on biogenesis, miRNA expression analysis is performed. Comparison of wild type and variant miRNA levels can be done at the level of the mature miRNA (Shen et al., 2009), assessing the total effect of the variant on the whole biogenesis process, or it can be done on several maturation levels of the miRNA, to deduce the exact step of the biogenesis that is affected by the variant (Duan et al., 2007).

If a variant is located in the mature miRNA sequence, targeting may be affected. Investigating which genes and pathways are affected due to the variant may provide insight in the disease mechanism by identifying new pathways and/or by confirming pathways previously hypothesized to be involved in the disease. Investigation and validation of direct interactions between miRNA and target RNAs can be done using reporter assays. Hereto, the potential binding site is cloned downstream of a reporter gene and cells are cotransfected with the miRNA and the reporter vector to assess whether the miRNA (or the variant miRNA) can target the site and thus reduce the reporter gene activity (Duan et al., 2007; Gong et al., 2012). While this allows confirmation of direct interactions, overexpression of the binding site and the miRNA may also lead to validation of interactions that may not take place endogenously in the cell type or tissue of interest, due to, for instance, absence of colocalization, or co-expression.

Investigation of variant induced targeting alterations can also be performed on a large scale by subjecting the wild type and variant miRNA expressing cells to transcriptome and/or proteome profiling. Single genes or proteins can be analyzed for differential expression. Pathway analysis can also identify which set of genes are influenced by the variant miRNA and may lead to biological meaningful findings about the variant miRNA and the pathways in the cell it affects in the disease state (Jazdzewski et al., 2009). If a variant is located outside the mature miRNA sequence, this approach can still be used if the variant affects mature miRNA expression, as the altered expression of the miRNA will also deregulate its target genes (Strazisar et al., 2015). However, in contrast to the reporter gene assays, these large-scale approaches cannot distinguish between direct and indirect targets of the miRNA, so target prediction algorithms need to be used to determine which of the deregulated genes can potentially be targeted directly by the miRNA.

Different crosslinking and immunoprecipitation (CLIP) approaches were developed and applied for global miRNA–RNA target interaction identification by Argonaute precipitation, such as HITS-CLIP and PAR-CLIP (Ule et al., 2005; Chi et al., 2009; Hafner et al., 2010). In these methods, RNA and proteins are crosslinked in cells by UV irradiation (in case of PAR-CLIP after incorporation of photoactivatable nucleosides). After cell lysis and partial RNA digestion, Argonaute-RNA complexes are pulled down and purified stringently. Subsequently, coprecipitated RNA is sequenced to identify which miRNAs and which target RNA regions were bound to Argonaute. Because miRNA and target RNAs are not physically linked to each other during the procedure, *in silico* target prediction is required to assess the interactions between the identified RNA sequences. These approaches thus provide enrichment for actual target RNA sites bound to Argonaute and limits the target prediction to these sites and to the detected miRNAs.

A method that can identify direct miRNA-target interactions on a large scale is the crosslinking, ligation, and sequencing of hybrids (CLASH) technology (Kudla et al., 2011; Helwak et al., 2013). Similar to the CLIP techniques, cells are UV irradiated to mediate crosslinking, after which Argonaute-RNA complexes are pulled down and RNA is partially digested. Then a ligation step is performed to ligate miRNAs to their target RNAs. The RNA is sequenced to identify chimeric RNAs. The non-chimeric reads provide the same information as the CLIP experiments, while the chimeric RNAs contain linked miRNA and target RNA fragments and hence provide direct proof of miRNA-target interactions. This technology may thus be used to directly identify whether mature miRNA variants alter targeting capacity of a disease-associated miRNA.

Together, these approaches can be used to assess what the effect of the genetic variant is on the expression and the functionality of the miRNA.

## Concluding Remarks

In this review we summarized the current knowledge of the miRNA maturation process, focusing on the prerequisites of the miRNA transcript to be processed. From these requirements it is clear that the RNA structure, and thus also the underlying sequence determining the structure, are of paramount importance for maturation into mature miRNAs. Thus genetic variants in miRNA genes can have large effects, not only on the miRNA gene itself, but due to its role as a fine-tuner of gene expression and translation, also on its downstream targets. In line with this, different miRNA variants have been found to be involved or hypothesized to be involved in human disease. We also highlighted the biological relevance of genetic variants located within or near miRNA genes and provided an overview of *in silico* and experimental approaches to investigate the effect of these variants on miRNA expression and function. This in turn could then be correlated to changes in miRNA and target gene abundance in the affected tissue of patients, as a first step in the understanding of the role of the miRNA in the disease pathomechanism.

As we are now coming to realize that the miRNA repertoire is much more complex than previously appreciated with the recognition of the wide-spread presence of isomiRs and their functional relevance, the possibilities of intronic miRNAs being transcribed independently from their host genes and even alternative splicing, many more questions arise. Though many aspects of miRNA biogenesis and functioning have already been unraveled, more investigations will need to be done to be able to fully grasp the regulation and the genesis of the correct expression pattern of the full miRNA repertoire in the cell. We also anticipate that, especially for the investigation of genetic variants in disease, we will need to undertake comprehensive and integrative approaches to be able to fully appreciate how certain genetic variants can undermine this regulatory system and lead to disease, while others remain completely harmless.

## Author Contributions

All authors contributed to the conception of the work. SC wrote the manuscript. MS, PD, and JD provided input and revised the manuscript. All authors approved the final manuscript.

## Conflict of Interest Statement

The authors declare that the research was conducted in the absence of any commercial or financial relationships that could be construed as a potential conflict of interest.

## Footnotes

1 http://mirvas.bioinf.be/
